# The layer silicate Cs_2_Sn^IV^Si_6_O_15_

**DOI:** 10.1107/S2056989021013554

**Published:** 2022-01-07

**Authors:** Michael Ketter, Matthias Weil

**Affiliations:** a Institute for Chemical Technologies and Analytics, Division of Structural Chemistry, TU Wien, Getreidemarkt 9/164-SC, A-1060 Vienna, Austria

**Keywords:** crystal structure, group-subgroup relation, caesium, tin, silicates

## Abstract

Single crystals of Cs_2_SnSi_6_O_15_, dicaesium tin(IV) hexa­silicate, were serendipitously obtained from a CsCl/NaCl flux at 923 K, starting from mixtures of CaO, SnO and TeO_2_ in a closed silica ampoule. The crystal structure of Cs_2_SnSi_6_O_15_ is constructed from {Si_6_O_15_}^6–^ layers extending parallel to (101), and Cs^I^ cations with a coordination number of eleven as well as isolated [SnO_6_] octa­hedra situated between the silicate layers. Each of the nine different SiO_4_ tetra­hedra in the silicate layer has a connectedness of *Q* ^3^ (three bridging and one terminal O atom), which leads to the formation of five- and eight-membered rings. The same type of silicate layer is found in the crystal structure of the mineral zeravshanite. Comparison with other silicates of the type Cs_2_ *M* ^IV^Si_6_O_15_ (*M* ^IV^ = Ti, Zr, Th, U) revealed a *klassengleiche* group–subgroup relationship of index 2 between Cs_2_ZrSi_6_O_15_ (*Z* = 6, space group *C*2/*m*) and Cs_2_SnSi_6_O_15_ (*Z* = 12, space group *I*2/*c*).

## Abstract 2

The crystal structure of Cs_2_SnSi_6_O_15_ shows a *klassengleiche* group–subgroup relationship of index 2 with Cs_2_ZrSi_6_O_15_.

## Chemical context

Calcium oxotellurate(IV), CaTeO_3_, is known to exist in various polymorphic forms that can be obtained either through solid-state reactions (Trömel & Ziethen-Reichenach, 1970 ▸; Stöger *et al.*, 2009 ▸) or under hydro­thermal conditions and subsequent dehydration (Poupon *et al.*, 2015 ▸). Some of the CaTeO_3_ polymorphs have a non-centrosymmetric crystal structure and show ferroelectric behaviour (Rai *et al.*, 2002 ▸) or a second harmonic generation (SHG) effect (Poupon *et al.*, 2015 ▸). These features are thought to be related to the presence of the 5*s* ^2^ electron lone pair at the Te^IV^ atom. In a current study it was attempted to incorporate Sn^II^ into CaTeO_3_ under formation of solid solutions (Ca_1-*x*_Sn_*x*_)TeO_3_ in order to investigate whether the 5*s* ^2^ electron lone pair at the Sn^II^ atom has any influence on the ferroelectric or SHG characteristics. For that purpose, a flux medium consisting of a eutectic CsCl/NaCl mixture with a melting point of 766 K (Żemcżużny & Rambach, 1909 ▸) was chosen as reaction medium in a closed silica ampoule. In comparison with conventional ceramic routes, this allows the lowering of the reaction temperatures by a simultaneous increase of the diffusion pathways. However, neither the intended (Ca_1-*x*_Sn_*x*_)TeO_3_ solid solutions nor other calcium oxotellurates with a partial replacement of Ca^II^ by Sn^II^ could be prepared this way. One of the side products of this reaction was Cs_2_Sn^IV^Si_6_O_15_, which had formed through attack of the silica glass ampoule by the molten salt mixture and concomitant oxidation of Sn^II^ to Sn^IV^. Its crystal structure along with a structural comparison with other silicates is given here.

## Structural commentary

**Figure 1:**
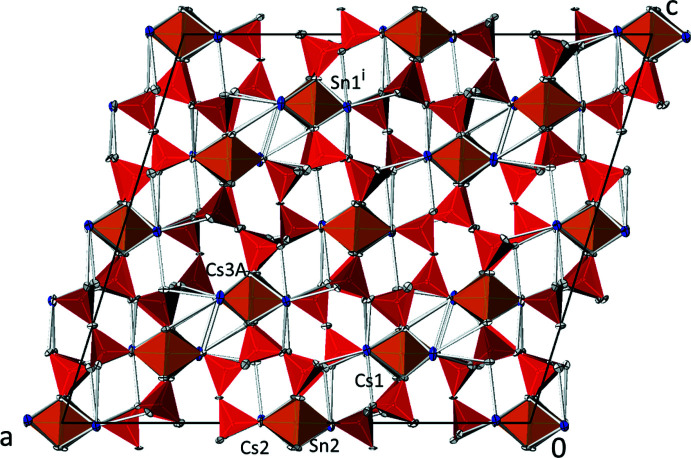
The crystal structure of Cs_2_SnSi_6_O_15_ in a projection along [0 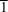 0]. Cs sites are given in blue, [SnO_6_] octa­hedra in orange and SiO_4_ tetra­hedra in red. Displacement parameters are drawn at the 74% probability level. For clarity, the disordered Cs3 site with minor occupancy (Cs3*B*) is not shown. [Symmetry code: (i) *x*, *y* + 1, *z* + 1.]

The asymmetric unit of Cs_2_SnSi_6_O_15_ comprises three Cs, two Sn, nine Si and twenty-three O sites. Except for one Sn site (Sn2) and one O site (O23), which are located on Wyckoff positions 4*b* (site symmetry 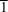 ) and 4*e* (site symmetry 2), respectively, all atoms are on general positions. The crystal structure of Cs_2_SnSi_6_O_15_ can be described as being built up from silicate layers extending parallel to (101). The silicate layers are linked by caesium cations and isolated [SnO_6_] octa­hedra situated between adjacent silicate layers (Fig. 1 ▸).

Each of the three caesium cations exhibits a coordination number of 11, with Cs—O bond lengths ranging from 2.951 (3) to 3.748 (3) Å. The mean Cs—O bond lengths for the three individual [CsO_11_] polyhedra (Cs1: 3.312 Å; Cs2: 3.355 Å; Cs3: 3.342 Å) are in very good agreement with the overall mean Cs—O bond length of 3.333 (226) Å calculated from 748 bonds for 11-coordinate Cs (Gagné & Hawthorne, 2016 ▸). The two Sn^IV^ atoms show slight deviations from a regular octa­hedral coordination, with Sn—O bond lengths ranging from 2.031 (3) to 2.058 (3) Å. The overall mean Sn^IV^—O bond length of 2.054 (10) Å calculated from 32 bonds (Gagné & Hawthorne, 2018 ▸) is somewhat longer than the mean values for Sn1 (2.037 Å) and Sn2 (2.047 Å).

**Figure 2:**
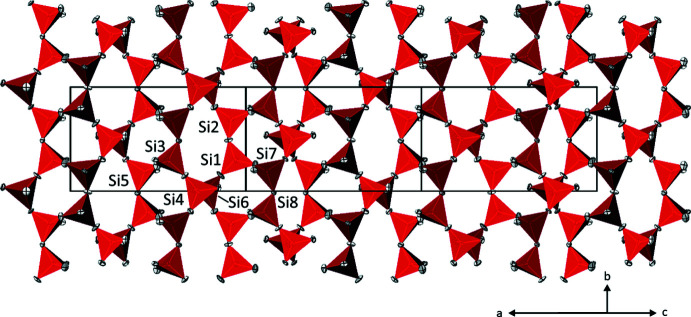
The {Si_6_O_15_}^6–^ layer in the crystal structure of Cs_2_SnSi_6_O_15_ shown in a projection along [ 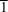 0 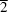 ]. Colour code and displacement ellipsoids are as in Fig.1.

All of the nine SiO_4_ tetra­hedra in the {Si_6_O_15_}^6–^ layer have one terminal O atom and are linked to three other SiO_4_ tetra­hedra by common bridging O atoms. Thus, the connectedness of the silicate tetra­hedra is Q^3^ according to the notation of Liebau (1985 ▸). The bond lengths distribution in the SiO_4_ tetra­hedra reflects the different roles of the O atoms in the silicate layer. The Si—O bonds to terminal O atoms are shorter by about 0.03 Å (mean 1.588 Å) than those to bridging O atoms (1.621 Å). The total mean Si—O bond in Cs_2_SnSi_6_O_15_ has a value of 1.613 Å, which is slightly shorter than the overall mean of 1.625 (24) calculated from 9128 bonds (Gagné & Hawthorne, 2018 ▸). The connectedness of the SiO_4_ tetra­hedra leads to the formation of a {Si_6_O_15_}^6–^ layer comprising five- and eight-membered rings (Fig. 2 ▸). The same type of silicate layer is found in the mineral zeravshanite with composition (Cs_3.80_Na_0.18_K_0.02_)Na_2_(Zr_2.73_Ti_0.19_Sn_0.04_Fe_0.04_)(Si_18_O_45_)(H_2_O)_2_ (Uvarova *et al.*, 2004 ▸).

Crystal-chemical features of silicates comprising the {Si_6_O_15_}^6–^ anion were recently compiled by Wierzbicka-Wieczorek *et al.* (2015 ▸). A topological classification of zirconosilicates and their analogues, where the simplest structure units are [*M*O_6_] octa­hedra and *T*O_4_ tetra­hedra united by vertices, was reported some time ago by Ilyushin & Blatov (2002 ▸). Since the same structure elements are also present in Cs_2_SnSi_6_O_15_ in the form of [SnO_6_] octa­hedra and SiO_4_ tetra­hedra, a similar analysis can be made. With respect to the concept of the polyhedral microensemble (PME) introduced by Ilyushin & Blatov (2002 ▸), Cs_2_SnSi_6_O_15_ conforms to the PME type C-1. A comparison of the unit-cell parameters of Cs_2_SnSi_6_O_15_ with those of the other reported Cs_2_ *M* ^IV^Si_6_O_15_ (*M* ^IV^ = Ti, Zr, Th, U) compounds (Table 1 ▸) revealed a close relationship between the Sn- and Zr-containing phases. The *a* and *b* axes and the *β* angle in the two structures are very similar, and the *c* axis of the Sn-containing compound is doubled. Indeed, there is a group–subgroup relationship between the crystal structures of Cs_2_ZrSi_6_O_15_ and Cs_2_SnSi_6_O_15_. The Sn-containing phase crystallizes in a subgroup (*I*2/*c*; *Z* = 12) of the Zr-containing phase (*C*2/*m*; *Z* = 6), defining a *klassengleiche* relationship of index 2 (Müller, 2013 ▸).

## Synthesis and crystallization

1.2 mmol of CaO (0.067 g), 0.13 mmol SnO (0.018 g) and 1.3 mmol of TeO_2_ (0.207 g) were intimately mixed with 1 g of an NaCl (35 mol%):CsCl (65 mol%) mixture and filled in a silica ampoule that was subsequently evacuated and torch-sealed. The ampoule was then heated at 923 K for 2 d before the power of the furnace was switched off. The silica ampoule showed a severe attack from the halide flux but was still intact. After washing the recrystallized flux with several portions of warm water and drying the remaining solid in air, a few lath-shaped crystals of the title compound could be isolated under a polarizing microscope. Single-crystal diffraction of other selected crystals revealed *α*-CaTeO_3_ (Stöger *et al.*, 2009 ▸), CaTe_2_O_5_ (Weil & Stöger, 2008 ▸) and Ca_4_Te_5_O_14_ (Weil, 2004 ▸) as products. Powder X-ray diffraction of the bulk showed the reflections of these phases together with those of SnO_2_ and also some reflections of non-assignable phase(s).

## Refinement

Crystal data, data collection and structure refinement details are summarized in Table 2 ▸. For better comparison of Cs_2_SnSi_6_O_15_ with the crystal structure of Cs_2_ZrSi_6_O_15_, the unconventional setting *I*2/*c* of space group type *C*2/*c* (No. 15) was chosen, so that unit-cell parameters *a*, *b*, *c* and *β* of the Sn-containing phase correspond to *a*, *b*, 2*c* and *β* of the Zr-containing phase (Jolicart *et al.*, 1996 ▸; Table 1 ▸). The Cs3 atom in Cs_2_SnSi_6_O_15_ was found to be disordered over two sites in a 0.934 (5):0.066 ratio and was refined with common displacement parameters for the two sites (*A* and *B*).

## Supplementary Material

Crystal structure: contains datablock(s) I. DOI: 10.1107/S2056989021013554/pk2660sup1.cif

Structure factors: contains datablock(s) I. DOI: 10.1107/S2056989021013554/pk2660Isup2.hkl

CCDC reference: 2130669

Additional supporting information:  crystallographic information; 3D view; checkCIF report

## Figures and Tables

**Table 1:** Crystal data (Å, °) of Cs_2_ *M* ^IV^Si_6_O_15_ compounds

*M*	Ti (single-crystal data)*^*a*^*	Ti (powder data)*^*b*^*	Zr*^*c*^*	Th (173 K data)*^*d*^*	Th (293 K data)*^*e*^*	U*^*f*^*
Space group	*C*2/*c*	*Cc*	*C*2/*m*	*Pca*2_1_	*Cmc*2_1_	*Cmc*2_1_
*Z*	4	4	6	4	4	4
*a*	13.386 (5)	12.988 (2)	26.610 (10)	16.2920 (10)	7.2813 (15)	7.2717 (3)
*b*	7.423 (3)	7.5014 (3)	7.506 (2)	7.2154 (6)	16.420 (3)	16.3061 (7)
*c*	15.134 (5)	15.156 (3)	11.602 (4)	13.6800 (10)	13.591 (3)	13.4983 (6)
*β*	107.71 (3)	105.80 (3)	107.43 (2)	90	90	90
*V*	1432.51	1420.83	2210.92	1608.13	1624.92	1600.53

References: (*a*) Grey *et al.* (1997 ▸); (*b*) Nyman *et al.* (2000 ▸); (*c*) Jolicart *et al.* (1996 ▸); (*d*) Woodward *et al.* (2005 ▸); (*e*) Mann *et al.* (2015 ▸); (*f*) Liu & Lii (2011 ▸).

**Table 2:** Experimental details

Crystal data	
Chemical formula	Cs_2_SnSi_6_O_15_
*M*_r_	793.05
Crystal system, space group	Monoclinic, *I*2/*c*
Temperature (K)	296
*a*, *b*, *c* (Å)	26.3434 (10), 7.4805 (3), 22.9137 (7)
β (°)	107.4020 (7)
*V* (Å^3^)	4308.7 (3)
*Z*	12
Radiation type	Mo *K*α
μ (mm^−1^)	7.36
Crystal size (mm)	0.12 × 0.03 × 0.01

Data collection	
Diffractometer	Bruker APEXII CCD
Absorption correction	Multi-scan (*SADABS*; Krause *et al.*, 2015 ▸).
*T*_min_, *T*_max_	0.539, 0.747
No. of measured, independent and observed [*I* > 2σ(*I*)] reflections	51032, 8282, 5013
*R*_int_	0.077
(sin θ/λ)_max_ (Å^−1^)	0.772

Refinement	
*R*[*F* ^2^ > 2σ(*F* ^2^)], *wR*(*F* ^2^), *S*	0.037, 0.082, 1.00
No. of reflections	8282
No. of parameters	331
Δρ_max_, Δρ_min_ (e Å^−3^)	1.84, −1.48

Computer programs: *APEX3* and *SAINT* (Bruker, 2018 ▸), *SHELXT* (Sheldrick, 2015*a* ▸), *SHELXL2018/3* (Sheldrick, 2015*b* ▸), *ATOMS* (Dowty, 2006 ▸) and *publCIF* (Westrip, 2010 ▸).

## supplementary crystallographic information

### Crystal data

**Table:**

Cs_2_SnSi_6_O_15_	*F*(000) = 4368
*M**_r_* = 793.05	*D*_x_ = 3.668 Mg m^−^^3^
Monoclinic, *I*2/*c*	Mo *K*α radiation, λ = 0.71073 Å
*a* = 26.3434 (10) Å	Cell parameters from 4399 reflections
*b* = 7.4805 (3) Å	θ = 2.8–32.4°
*c* = 22.9137 (7) Å	µ = 7.36 mm^−^^1^
β = 107.4020 (7)°	*T* = 296 K
*V* = 4308.7 (3) Å^3^	Lath, colourless
*Z* = 12	0.12 × 0.03 × 0.01 mm

### Data collection

**Table:**

Bruker APEXII CCD diffractometer	5013 reflections with *I* > 2σ(*I*)
ω– and φ–scans	*R*_int_ = 0.077
Absorption correction: multi-scan (*SADABS*; Krause *et al.*, 2015).	θ_max_ = 33.3°, θ_min_ = 2.8°
*T*_min_ = 0.539, *T*_max_ = 0.747	*h* = −40→40
51032 measured reflections	*k* = −11→11
8282 independent reflections	*l* = −35→35

### Refinement

**Table:**

Refinement on *F*^2^	331 parameters
Least-squares matrix: full	0 restraints
*R*[*F*^2^ > 2σ(*F*^2^)] = 0.037	*w* = 1/[σ^2^(*F*_o_^2^) + (0.0259*P*)^2^] where *P* = (*F*_o_^2^ + 2*F*_c_^2^)/3
*wR*(*F*^2^) = 0.082	(Δ/σ)_max_ = 0.002
*S* = 1.00	Δρ_max_ = 1.84 e Å^−^^3^
8282 reflections	Δρ_min_ = −1.48 e Å^−^^3^

### Special details

**Table:**

Geometry. All esds (except the esd in the dihedral angle between two l.s. planes) are estimated using the full covariance matrix. The cell esds are taken into account individually in the estimation of esds in distances, angles and torsion angles; correlations between esds in cell parameters are only used when they are defined by crystal symmetry. An approximate (isotropic) treatment of cell esds is used for estimating esds involving l.s. planes.

### Fractional atomic coordinates and isotropic or equivalent isotropic displacement parameters (Å^2^)

**Table:**

	*x*	*y*	*z*	*U*_iso_*/*U*_eq_	Occ. (<1)
Cs1	0.39755 (2)	−0.01953 (4)	0.18693 (2)	0.01982 (7)	
Cs2	0.57484 (2)	−0.51580 (4)	0.00767 (2)	0.02128 (7)	
Cs3A	0.74609 (2)	−0.98408 (7)	0.32160 (8)	0.02213 (18)	0.934 (5)
Cs3B	0.7514 (3)	−0.9776 (11)	0.3439 (8)	0.02213 (18)	0.066 (5)
Sn1	0.67822 (2)	−0.49735 (4)	−0.17784 (2)	0.00700 (6)	
Sn2	0.500000	0.000000	0.000000	0.00681 (7)	
Si1	0.62500 (5)	−0.70216 (15)	0.18853 (6)	0.0075 (2)	
Si2	0.63425 (5)	−0.30679 (15)	0.18719 (6)	0.0091 (2)	
Si3	0.69676 (5)	−0.70648 (15)	−0.04744 (6)	0.0077 (2)	
Si4	0.63589 (4)	−0.99872 (15)	−0.00070 (5)	0.0067 (2)	
Si5	0.81053 (5)	−0.81223 (15)	0.04445 (6)	0.0086 (2)	
Si6	0.68624 (4)	−0.99635 (15)	0.13636 (5)	0.0078 (2)	
Si7	0.50782 (5)	−0.80838 (15)	0.13301 (6)	0.0080 (2)	
Si8	0.51736 (5)	−0.20099 (15)	0.13222 (6)	0.0081 (2)	
Si9	0.45804 (4)	−0.50114 (15)	0.18261 (5)	0.0066 (2)	
O1	0.65897 (13)	−0.7024 (4)	0.25901 (15)	0.0129 (7)	
O2	0.56382 (13)	−0.7463 (4)	0.18108 (15)	0.0167 (7)	
O3	0.64737 (13)	−0.8469 (4)	0.15066 (16)	0.0199 (8)	
O4	0.62794 (13)	−0.5077 (4)	0.15681 (13)	0.0140 (6)	
O5	0.66980 (12)	−0.3096 (4)	0.25683 (15)	0.0115 (6)	
O6	0.66307 (13)	−0.1918 (4)	0.14653 (15)	0.0162 (7)	
O7	0.57564 (13)	−0.2302 (4)	0.18125 (15)	0.0170 (7)	
O8	0.70020 (13)	−0.6982 (4)	−0.11572 (15)	0.0119 (7)	
O9	0.75309 (13)	−0.7544 (5)	0.00067 (17)	0.0187 (7)	
O10	0.65155 (12)	−0.8509 (4)	−0.04422 (15)	0.0134 (7)	
O11	0.67790 (13)	−0.5157 (4)	−0.02671 (14)	0.0144 (6)	
O12	0.57747 (12)	−0.9680 (4)	0.00220 (15)	0.0144 (7)	
O13	0.68113 (12)	−0.9797 (4)	0.06475 (13)	0.0123 (6)	
O14	0.85664 (13)	−0.6934 (4)	0.02893 (16)	0.0147 (7)	
O15	0.81334 (13)	−0.7987 (4)	0.11477 (14)	0.0125 (7)	
O16	0.74525 (12)	−0.9636 (4)	0.17887 (14)	0.0136 (7)	
O17	0.50824 (13)	−0.7996 (4)	0.06370 (15)	0.0127 (7)	
O18	0.46185 (12)	−0.6903 (4)	0.14849 (15)	0.0129 (7)	
O19	0.49642 (12)	−0.0117 (4)	0.15154 (14)	0.0119 (6)	
O20	0.51978 (13)	−0.1991 (4)	0.06375 (15)	0.0121 (7)	
O21	0.47656 (13)	−0.3514 (4)	0.14152 (16)	0.0171 (7)	
O22	0.39930 (12)	−0.4614 (4)	0.18260 (15)	0.0149 (7)	
O23	0.500000	−0.5021 (6)	0.250000	0.0193 (10)	

### Atomic displacement parameters (Å^2^)

**Table:**

	*U*^11^	*U*^22^	*U*^33^	*U*^12^	*U*^13^	*U*^23^
Cs1	0.01741 (15)	0.02075 (15)	0.02457 (17)	−0.00118 (12)	0.01126 (13)	−0.00080 (13)
Cs2	0.01832 (15)	0.02304 (15)	0.02465 (17)	−0.00150 (14)	0.00974 (13)	−0.00107 (14)
Cs3A	0.01454 (17)	0.02048 (16)	0.0320 (5)	0.00139 (14)	0.0080 (2)	0.0013 (2)
Cs3B	0.01454 (17)	0.02048 (16)	0.0320 (5)	0.00139 (14)	0.0080 (2)	0.0013 (2)
Sn1	0.00705 (12)	0.00726 (12)	0.00620 (13)	0.00027 (11)	0.00124 (10)	0.00007 (12)
Sn2	0.00707 (17)	0.00700 (17)	0.00653 (18)	0.00038 (16)	0.00229 (14)	0.00016 (17)
Si1	0.0074 (6)	0.0074 (5)	0.0069 (6)	−0.0006 (4)	0.0011 (5)	−0.0018 (4)
Si2	0.0098 (6)	0.0086 (5)	0.0082 (7)	−0.0011 (4)	0.0013 (5)	0.0012 (4)
Si3	0.0086 (6)	0.0076 (5)	0.0064 (7)	0.0007 (4)	0.0016 (5)	0.0012 (4)
Si4	0.0063 (5)	0.0065 (5)	0.0079 (5)	−0.0005 (4)	0.0031 (4)	0.0001 (5)
Si5	0.0093 (6)	0.0071 (5)	0.0091 (7)	0.0004 (4)	0.0021 (5)	0.0010 (4)
Si6	0.0077 (5)	0.0091 (5)	0.0065 (5)	0.0003 (5)	0.0020 (4)	−0.0002 (5)
Si7	0.0086 (6)	0.0063 (5)	0.0091 (7)	−0.0002 (4)	0.0025 (5)	−0.0012 (4)
Si8	0.0089 (6)	0.0072 (5)	0.0086 (7)	0.0011 (4)	0.0031 (5)	0.0020 (4)
Si9	0.0068 (5)	0.0064 (5)	0.0071 (5)	−0.0012 (5)	0.0029 (4)	−0.0004 (5)
O1	0.0160 (17)	0.0111 (15)	0.0096 (18)	0.0011 (12)	0.0008 (14)	−0.0006 (12)
O2	0.0086 (16)	0.0259 (18)	0.0147 (19)	−0.0032 (13)	0.0020 (14)	−0.0048 (14)
O3	0.0168 (18)	0.0198 (17)	0.022 (2)	0.0065 (14)	0.0036 (16)	−0.0121 (14)
O4	0.0229 (17)	0.0077 (13)	0.0097 (15)	−0.0037 (13)	0.0026 (13)	−0.0007 (12)
O5	0.0119 (16)	0.0099 (14)	0.0110 (18)	−0.0009 (12)	0.0011 (14)	0.0037 (12)
O6	0.0219 (19)	0.0129 (15)	0.0154 (19)	−0.0034 (13)	0.0079 (16)	0.0028 (13)
O7	0.0102 (17)	0.0242 (18)	0.0131 (19)	0.0048 (13)	−0.0019 (14)	0.0041 (14)
O8	0.0164 (17)	0.0092 (14)	0.0105 (18)	0.0016 (12)	0.0049 (14)	0.0024 (12)
O9	0.0121 (16)	0.0264 (17)	0.0146 (17)	0.0030 (13)	−0.0005 (13)	0.0072 (13)
O10	0.0129 (17)	0.0145 (15)	0.0105 (17)	−0.0063 (12)	0.0001 (14)	0.0045 (12)
O11	0.0209 (17)	0.0096 (14)	0.0175 (17)	−0.0002 (13)	0.0131 (14)	−0.0014 (13)
O12	0.0071 (15)	0.0177 (15)	0.0203 (18)	−0.0010 (12)	0.0068 (13)	−0.0010 (13)
O13	0.0134 (15)	0.0193 (16)	0.0030 (14)	−0.0020 (13)	0.0006 (12)	0.0005 (12)
O14	0.0124 (17)	0.0131 (15)	0.018 (2)	−0.0025 (12)	0.0042 (15)	0.0036 (13)
O15	0.0196 (18)	0.0097 (14)	0.0070 (17)	0.0016 (12)	0.0022 (14)	0.0007 (12)
O16	0.0083 (15)	0.0237 (17)	0.0081 (16)	−0.0012 (12)	0.0013 (12)	−0.0024 (13)
O17	0.0195 (18)	0.0111 (14)	0.0072 (18)	−0.0012 (13)	0.0036 (14)	−0.0039 (12)
O18	0.0117 (16)	0.0096 (14)	0.0178 (19)	0.0030 (12)	0.0052 (14)	−0.0048 (12)
O19	0.0146 (15)	0.0106 (14)	0.0132 (15)	0.0011 (13)	0.0084 (13)	0.0002 (13)
O20	0.0162 (17)	0.0106 (14)	0.0107 (18)	0.0033 (12)	0.0060 (14)	0.0009 (12)
O21	0.0201 (19)	0.0143 (16)	0.017 (2)	−0.0077 (13)	0.0064 (16)	0.0061 (13)
O22	0.0081 (15)	0.0155 (15)	0.0211 (18)	0.0013 (12)	0.0042 (14)	−0.0025 (13)
O23	0.020 (2)	0.025 (2)	0.011 (2)	0.000	0.0024 (19)	0.000

### Geometric parameters (Å, º)

**Table:**

Cs1—O19	2.951 (3)	Sn1—O22^iii^	2.036 (3)
Cs1—O1^i^	3.237 (3)	Sn1—O1^x^	2.036 (3)
Cs1—O18^ii^	3.256 (3)	Sn1—O15^vi^	2.038 (3)
Cs1—O10^iii^	3.282 (3)	Sn1—O16^vi^	2.039 (3)
Cs1—O7^iv^	3.291 (3)	Sn1—O5^x^	2.044 (3)
Cs1—O5^iv^	3.299 (3)	Sn2—O12^iii^	2.040 (3)
Cs1—O22	3.308 (3)	Sn2—O12^ii^	2.040 (3)
Cs1—O15^v^	3.336 (3)	Sn2—O20	2.042 (3)
Cs1—O8^iii^	3.352 (3)	Sn2—O20^xi^	2.042 (3)
Cs1—O2^i^	3.538 (3)	Sn2—O17^iii^	2.058 (3)
Cs1—O21	3.588 (3)	Sn2—O17^ii^	2.058 (3)
Cs1—Cs3A^i^	3.7396 (5)	Si1—O1	1.595 (4)
Cs2—O11	3.045 (3)	Si1—O2	1.604 (3)
Cs2—O20	3.238 (3)	Si1—O3	1.606 (3)
Cs2—O17	3.253 (3)	Si1—O4	1.638 (3)
Cs2—O14^vi^	3.268 (3)	Si2—O5	1.590 (3)
Cs2—O4	3.283 (3)	Si2—O7	1.614 (3)
Cs2—O17^iii^	3.300 (3)	Si2—O6	1.615 (3)
Cs2—O20^iii^	3.316 (3)	Si2—O4	1.644 (3)
Cs2—O12	3.387 (3)	Si3—O8	1.595 (3)
Cs2—O21^iii^	3.426 (4)	Si3—O9	1.602 (4)
Cs2—O10	3.636 (3)	Si3—O10	1.626 (3)
Cs2—O18^iii^	3.748 (3)	Si3—O11	1.628 (3)
Cs2—Cs2^iii^	3.8598 (6)	Si4—O12	1.577 (3)
Cs3A—O1	3.129 (3)	Si4—O13	1.619 (3)
Cs3A—O8^vii^	3.195 (3)	Si4—O10	1.623 (3)
Cs3A—O4^viii^	3.206 (3)	Si4—O14^xii^	1.629 (3)
Cs3A—O5^ix^	3.223 (3)	Si5—O15	1.594 (3)
Cs3A—O15^viii^	3.227 (3)	Si5—O9	1.605 (4)
Cs3A—O16	3.268 (3)	Si5—O11^xii^	1.628 (3)
Cs3A—O6^viii^	3.331 (3)	Si5—O14	1.628 (3)
Cs3A—O16^viii^	3.357 (3)	Si6—O16	1.587 (3)
Cs3A—Si2^viii^	3.5783 (13)	Si6—O13	1.611 (3)
Cs3A—O5^viii^	3.592 (3)	Si6—O3	1.615 (3)
Cs3A—O3^viii^	3.653 (3)	Si6—O6^ix^	1.628 (3)
Cs3B—O15^viii^	3.007 (10)	Si7—O17	1.593 (3)
Cs3B—O8^vii^	3.050 (9)	Si7—O18	1.622 (3)
Cs3B—O4^viii^	3.186 (8)	Si7—O2	1.623 (4)
Cs3B—O6^viii^	3.308 (8)	Si7—O19^ix^	1.631 (3)
Cs3B—O1	3.334 (12)	Si8—O20	1.589 (3)
Cs3B—O16^viii^	3.347 (9)	Si8—O21	1.614 (3)
Cs3B—O5^ix^	3.494 (13)	Si8—O7	1.623 (4)
Cs3B—O3^viii^	3.580 (8)	Si8—O19	1.629 (3)
Cs3B—Si2^viii^	3.670 (8)	Si9—O22	1.576 (3)
Cs3B—O16	3.738 (18)	Si9—O23	1.6078 (12)
Cs3B—Si1^viii^	3.796 (8)	Si9—O21	1.629 (3)
Sn1—O8	2.031 (3)	Si9—O18	1.634 (3)
			
O19—Cs1—O1^i^	129.44 (8)	O1—Si1—Cs3A^viii^	79.54 (12)
O19—Cs1—O18^ii^	48.50 (7)	O2—Si1—Cs3A^viii^	166.44 (14)
O1^i^—Cs1—O18^ii^	83.41 (7)	O3—Si1—Cs3A^viii^	74.38 (13)
O19—Cs1—O10^iii^	81.34 (8)	O4—Si1—Cs3A^viii^	58.51 (12)
O1^i^—Cs1—O10^iii^	120.55 (8)	O1—Si1—Cs3B^viii^	86.8 (3)
O18^ii^—Cs1—O10^iii^	92.05 (8)	O2—Si1—Cs3B^viii^	161.0 (3)
O19—Cs1—O7^iv^	107.28 (8)	O3—Si1—Cs3B^viii^	70.0 (2)
O1^i^—Cs1—O7^iv^	89.80 (8)	O4—Si1—Cs3B^viii^	56.05 (18)
O18^ii^—Cs1—O7^iv^	128.63 (8)	Cs3A^viii^—Si1—Cs3B^viii^	7.4 (2)
O10^iii^—Cs1—O7^iv^	133.27 (8)	O1—Si1—Cs1^xiii^	54.50 (11)
O19—Cs1—O5^iv^	137.94 (8)	O2—Si1—Cs1^xiii^	65.38 (13)
O1^i^—Cs1—O5^iv^	88.26 (7)	O3—Si1—Cs1^xiii^	98.41 (13)
O18^ii^—Cs1—O5^iv^	170.79 (7)	O4—Si1—Cs1^xiii^	154.79 (12)
O10^iii^—Cs1—O5^iv^	95.58 (7)	Cs3A^viii^—Si1—Cs1^xiii^	127.81 (4)
O7^iv^—Cs1—O5^iv^	47.11 (8)	Cs3B^viii^—Si1—Cs1^xiii^	133.4 (2)
O19—Cs1—O22	89.45 (8)	O1—Si1—Cs2	156.41 (12)
O1^i^—Cs1—O22	139.09 (7)	O2—Si1—Cs2	87.35 (13)
O18^ii^—Cs1—O22	137.29 (7)	O3—Si1—Cs2	75.41 (14)
O10^iii^—Cs1—O22	71.33 (8)	O4—Si1—Cs2	46.31 (11)
O7^iv^—Cs1—O22	63.12 (8)	Cs3A^viii^—Si1—Cs2	80.71 (3)
O5^iv^—Cs1—O22	50.83 (7)	Cs3B^viii^—Si1—Cs2	74.0 (3)
O19—Cs1—O15^v^	111.12 (8)	Cs1^xiii^—Si1—Cs2	148.79 (3)
O1^i^—Cs1—O15^v^	49.69 (8)	O1—Si1—Cs3A	36.91 (11)
O18^ii^—Cs1—O15^v^	69.44 (7)	O2—Si1—Cs3A	119.24 (13)
O10^iii^—Cs1—O15^v^	73.23 (8)	O3—Si1—Cs3A	74.66 (14)
O7^iv^—Cs1—O15^v^	136.47 (7)	O4—Si1—Cs3A	129.29 (12)
O5^iv^—Cs1—O15^v^	107.90 (7)	Cs3A^viii^—Si1—Cs3A	74.28 (3)
O22—Cs1—O15^v^	135.35 (8)	Cs3B^viii^—Si1—Cs3A	79.2 (2)
O19—Cs1—O8^iii^	119.44 (8)	Cs1^xiii^—Si1—Cs3A	54.428 (19)
O1^i^—Cs1—O8^iii^	105.87 (7)	Cs2—Si1—Cs3A	145.09 (3)
O18^ii^—Cs1—O8^iii^	136.95 (8)	O5—Si2—O7	110.53 (17)
O10^iii^—Cs1—O8^iii^	46.58 (7)	O5—Si2—O6	110.39 (18)
O7^iv^—Cs1—O8^iii^	93.90 (8)	O7—Si2—O6	111.42 (18)
O5^iv^—Cs1—O8^iii^	49.56 (8)	O5—Si2—O4	111.56 (16)
O22—Cs1—O8^iii^	51.07 (7)	O7—Si2—O4	108.41 (18)
O15^v^—Cs1—O8^iii^	84.72 (7)	O6—Si2—O4	104.37 (16)
O19—Cs1—O2^i^	99.87 (8)	O5—Si2—Cs3A^viii^	77.67 (12)
O1^i^—Cs1—O2^i^	45.36 (8)	O7—Si2—Cs3A^viii^	170.77 (14)
O18^ii^—Cs1—O2^i^	75.78 (8)	O6—Si2—Cs3A^viii^	68.05 (12)
O10^iii^—Cs1—O2^i^	161.62 (7)	O4—Si2—Cs3A^viii^	63.62 (12)
O7^iv^—Cs1—O2^i^	64.15 (7)	O5—Si2—Cs3B^viii^	85.3 (3)
O5^iv^—Cs1—O2^i^	95.55 (7)	O7—Si2—Cs3B^viii^	163.7 (3)
O22—Cs1—O2^i^	126.86 (8)	O6—Si2—Cs3B^viii^	64.3 (2)
O15^v^—Cs1—O2^i^	89.43 (7)	O4—Si2—Cs3B^viii^	60.1 (2)
O8^iii^—Cs1—O2^i^	139.69 (7)	Cs3A^viii^—Si2—Cs3B^viii^	7.6 (3)
O19—Cs1—O21	45.41 (7)	O5—Si2—Cs1^iv^	56.99 (11)
O1^i^—Cs1—O21	172.30 (8)	O7—Si2—Cs1^iv^	56.83 (13)
O18^ii^—Cs1—O21	92.93 (7)	O6—Si2—Cs1^iv^	112.15 (12)
O10^iii^—Cs1—O21	66.22 (8)	O4—Si2—Cs1^iv^	143.45 (12)
O7^iv^—Cs1—O21	87.19 (8)	Cs3A^viii^—Si2—Cs1^iv^	132.30 (5)
O5^iv^—Cs1—O21	94.85 (7)	Cs3B^viii^—Si2—Cs1^iv^	139.4 (3)
O22—Cs1—O21	44.39 (7)	O5—Si2—Cs2	154.76 (12)
O15^v^—Cs1—O21	135.03 (8)	O7—Si2—Cs2	89.01 (13)
O8^iii^—Cs1—O21	81.42 (7)	O6—Si2—Cs2	74.77 (13)
O2^i^—Cs1—O21	127.16 (8)	O4—Si2—Cs2	44.80 (11)
O19—Cs1—Cs3A^i^	161.21 (7)	Cs3A^viii^—Si2—Cs2	81.95 (4)
O1^i^—Cs1—Cs3A^i^	52.70 (6)	Cs3B^viii^—Si2—Cs2	74.7 (3)
O18^ii^—Cs1—Cs3A^i^	122.16 (5)	Cs1^iv^—Si2—Cs2	145.67 (4)
O10^iii^—Cs1—Cs3A^i^	82.98 (6)	O5—Si2—Cs3A^ii^	38.62 (11)
O7^iv^—Cs1—Cs3A^i^	91.03 (6)	O7—Si2—Cs3A^ii^	107.08 (13)
O5^iv^—Cs1—Cs3A^i^	54.06 (6)	O6—Si2—Cs3A^ii^	77.00 (13)
O22—Cs1—Cs3A^i^	95.29 (5)	O4—Si2—Cs3A^ii^	140.89 (12)
O15^v^—Cs1—Cs3A^i^	53.91 (5)	Cs3A^viii^—Si2—Cs3A^ii^	81.93 (4)
O8^iii^—Cs1—Cs3A^i^	53.22 (5)	Cs3B^viii^—Si2—Cs3A^ii^	87.7 (2)
O2^i^—Cs1—Cs3A^i^	91.81 (5)	Cs1^iv^—Si2—Cs3A^ii^	54.01 (2)
O21—Cs1—Cs3A^i^	134.39 (5)	Cs2—Si2—Cs3A^ii^	151.10 (3)
O11—Cs2—O20	129.98 (8)	O8—Si3—O9	111.77 (18)
O11—Cs2—O17	136.90 (8)	O8—Si3—O10	109.12 (18)
O20—Cs2—O17	87.84 (7)	O9—Si3—O10	111.11 (18)
O11—Cs2—O14^vi^	47.93 (7)	O8—Si3—O11	111.35 (16)
O20—Cs2—O14^vi^	84.79 (7)	O9—Si3—O11	107.22 (19)
O17—Cs2—O14^vi^	170.85 (8)	O10—Si3—O11	106.12 (16)
O11—Cs2—O4	97.70 (8)	O8—Si3—Cs1^iii^	55.75 (12)
O20—Cs2—O4	71.06 (8)	O9—Si3—Cs1^iii^	131.64 (14)
O17—Cs2—O4	73.34 (8)	O10—Si3—Cs1^iii^	53.45 (12)
O14^vi^—Cs2—O4	99.06 (8)	O11—Si3—Cs1^iii^	120.94 (13)
O11—Cs2—O17^iii^	112.47 (8)	O8—Si3—Cs3B^xiv^	43.6 (2)
O20—Cs2—O17^iii^	50.51 (7)	O9—Si3—Cs3B^xiv^	82.2 (2)
O17—Cs2—O17^iii^	107.82 (6)	O10—Si3—Cs3B^xiv^	92.32 (16)
O14^vi^—Cs2—O17^iii^	71.38 (8)	O11—Si3—Cs3B^xiv^	153.9 (2)
O4—Cs2—O17^iii^	121.04 (7)	Cs1^iii^—Si3—Cs3B^xiv^	56.65 (16)
O11—Cs2—O20^iii^	117.98 (7)	O8—Si3—Cs2	125.15 (13)
O20—Cs2—O20^iii^	107.85 (6)	O9—Si3—Cs2	121.49 (14)
O17—Cs2—O20^iii^	50.25 (7)	O10—Si3—Cs2	63.86 (12)
O14^vi^—Cs2—O20^iii^	137.65 (8)	O11—Si3—Cs2	42.26 (11)
O4—Cs2—O20^iii^	123.29 (7)	Cs1^iii^—Si3—Cs2	93.96 (3)
O17^iii^—Cs2—O20^iii^	85.76 (7)	Cs3B^xiv^—Si3—Cs2	150.41 (15)
O11—Cs2—O12	87.76 (7)	O8—Si3—Cs3A^xiv^	38.41 (11)
O20—Cs2—O12	139.65 (7)	O9—Si3—Cs3A^xiv^	87.05 (14)
O17—Cs2—O12	51.84 (7)	O10—Si3—Cs3A^xiv^	93.01 (12)
O14^vi^—Cs2—O12	135.12 (7)	O11—Si3—Cs3A^xiv^	149.35 (12)
O4—Cs2—O12	92.86 (8)	Cs1^iii^—Si3—Cs3A^xiv^	53.629 (19)
O17^iii^—Cs2—O12	135.53 (8)	Cs3B^xiv^—Si3—Cs3A^xiv^	5.5 (2)
O20^iii^—Cs2—O12	50.21 (7)	Cs2—Si3—Cs3A^xiv^	147.54 (4)
O11—Cs2—O21^iii^	81.88 (8)	O12—Si4—O13	113.92 (17)
O20—Cs2—O21^iii^	121.19 (8)	O12—Si4—O10	110.94 (17)
O17—Cs2—O21^iii^	96.07 (7)	O13—Si4—O10	104.91 (17)
O14^vi^—Cs2—O21^iii^	92.39 (8)	O12—Si4—O14^xii^	112.47 (17)
O4—Cs2—O21^iii^	164.18 (7)	O13—Si4—O14^xii^	107.67 (17)
O17^iii^—Cs2—O21^iii^	72.97 (8)	O10—Si4—O14^xii^	106.39 (17)
O20^iii^—Cs2—O21^iii^	46.12 (7)	O12—Si4—Cs2	56.81 (11)
O12—Cs2—O21^iii^	71.33 (8)	O13—Si4—Cs2	94.11 (11)
O11—Cs2—O10	44.74 (7)	O10—Si4—Cs2	65.92 (11)
O20—Cs2—O10	173.27 (8)	O14^xii^—Si4—Cs2	158.21 (14)
O17—Cs2—O10	95.59 (7)	O12—Si4—Cs1^iii^	97.51 (13)
O14^vi^—Cs2—O10	91.21 (7)	O13—Si4—Cs1^iii^	146.31 (11)
O4—Cs2—O10	104.34 (7)	O10—Si4—Cs1^iii^	49.59 (12)
O17^iii^—Cs2—O10	133.00 (7)	O14^xii^—Si4—Cs1^iii^	68.28 (12)
O20^iii^—Cs2—O10	78.73 (7)	Cs2—Si4—Cs1^iii^	93.20 (2)
O12—Cs2—O10	43.92 (6)	O12—Si4—Cs2^ix^	75.01 (11)
O21^iii^—Cs2—O10	64.29 (8)	O13—Si4—Cs2^ix^	102.93 (11)
O11—Cs2—O18^iii^	75.42 (8)	O10—Si4—Cs2^ix^	145.47 (12)
O20—Cs2—O18^iii^	93.42 (7)	O14^xii^—Si4—Cs2^ix^	44.83 (11)
O17—Cs2—O18^iii^	129.03 (8)	Cs2—Si4—Cs2^ix^	131.69 (3)
O14^vi^—Cs2—O18^iii^	57.05 (8)	Cs1^iii^—Si4—Cs2^ix^	96.43 (2)
O4—Cs2—O18^iii^	153.40 (7)	O15—Si5—O9	111.40 (18)
O17^iii^—Cs2—O18^iii^	44.71 (7)	O15—Si5—O11^xii^	110.88 (16)
O20^iii^—Cs2—O18^iii^	81.50 (7)	O9—Si5—O11^xii^	107.93 (19)
O12—Cs2—O18^iii^	112.20 (7)	O15—Si5—O14	111.96 (18)
O21^iii^—Cs2—O18^iii^	41.79 (7)	O9—Si5—O14	110.00 (18)
O10—Cs2—O18^iii^	88.92 (6)	O11^xii^—Si5—O14	104.38 (16)
O11—Cs2—Cs2^iii^	160.41 (6)	O15—Si5—Cs2^xii^	122.11 (13)
O20—Cs2—Cs2^iii^	54.87 (6)	O9—Si5—Cs2^xii^	126.10 (14)
O17—Cs2—Cs2^iii^	54.48 (6)	O11^xii^—Si5—Cs2^xii^	48.26 (11)
O14^vi^—Cs2—Cs2^iii^	123.98 (6)	O14—Si5—Cs2^xii^	56.37 (11)
O4—Cs2—Cs2^iii^	101.49 (5)	O15—Si5—Cs3B^viii^	39.9 (2)
O17^iii^—Cs2—Cs2^iii^	53.35 (5)	O9—Si5—Cs3B^viii^	76.6 (2)
O20^iii^—Cs2—Cs2^iii^	52.98 (5)	O11^xii^—Si5—Cs3B^viii^	143.06 (18)
O12—Cs2—Cs2^iii^	95.13 (5)	O14—Si5—Cs3B^viii^	108.28 (16)
O21^iii^—Cs2—Cs2^iii^	80.72 (5)	Cs2^xii^—Si5—Cs3B^viii^	154.58 (17)
O10—Cs2—Cs2^iii^	131.69 (5)	O15—Si5—Cs1^xv^	47.65 (11)
O18^iii^—Cs2—Cs2^iii^	85.59 (5)	O9—Si5—Cs1^xv^	121.39 (14)
O1—Cs3A—O8^vii^	112.49 (8)	O11^xii^—Si5—Cs1^xv^	130.40 (13)
O1—Cs3A—O4^viii^	131.83 (8)	O14—Si5—Cs1^xv^	64.99 (13)
O8^vii^—Cs3A—O4^viii^	115.45 (8)	Cs2^xii^—Si5—Cs1^xv^	100.22 (3)
O1—Cs3A—O5^ix^	91.53 (8)	Cs3B^viii^—Si5—Cs1^xv^	54.65 (16)
O8^vii^—Cs3A—O5^ix^	51.50 (8)	O16—Si6—O13	112.41 (16)
O4^viii^—Cs3A—O5^ix^	122.05 (8)	O16—Si6—O3	109.44 (18)
O1—Cs3A—O15^viii^	51.50 (8)	O13—Si6—O3	106.76 (18)
O8^vii^—Cs3A—O15^viii^	89.11 (8)	O16—Si6—O6^ix^	113.01 (18)
O4^viii^—Cs3A—O15^viii^	124.22 (9)	O13—Si6—O6^ix^	107.14 (17)
O5^ix^—Cs3A—O15^viii^	112.54 (8)	O3—Si6—O6^ix^	107.78 (18)
O1—Cs3A—O16	74.06 (8)	O16—Si6—Cs3B^viii^	59.0 (2)
O8^vii^—Cs3A—O16	126.48 (9)	O13—Si6—Cs3B^viii^	87.2 (3)
O4^viii^—Cs3A—O16	81.50 (8)	O3—Si6—Cs3B^viii^	67.37 (17)
O5^ix^—Cs3A—O16	75.98 (8)	O6^ix^—Si6—Cs3B^viii^	165.6 (3)
O15^viii^—Cs3A—O16	124.23 (8)	O16—Si6—Cs3A^viii^	55.21 (12)
O1—Cs3A—O6^viii^	166.14 (10)	O13—Si6—Cs3A^viii^	94.08 (11)
O8^vii^—Cs3A—O6^viii^	72.76 (8)	O3—Si6—Cs3A^viii^	65.97 (13)
O4^viii^—Cs3A—O6^viii^	46.33 (7)	O6^ix^—Si6—Cs3A^viii^	158.73 (13)
O5^ix^—Cs3A—O6^viii^	81.91 (8)	Cs3B^viii^—Si6—Cs3A^viii^	6.9 (2)
O15^viii^—Cs3A—O6^viii^	142.36 (10)	O16—Si6—Cs3A	49.66 (11)
O16—Cs3A—O6^viii^	92.45 (8)	O13—Si6—Cs3A	161.93 (12)
O1—Cs3A—O16^viii^	50.82 (8)	O3—Si6—Cs3A	81.01 (14)
O8^vii^—Cs3A—O16^viii^	141.13 (8)	O6^ix^—Si6—Cs3A	85.16 (13)
O4^viii^—Cs3A—O16^viii^	87.21 (7)	Cs3B^viii^—Si6—Cs3A	80.7 (3)
O5^ix^—Cs3A—O16^viii^	141.80 (8)	Cs3A^viii^—Si6—Cs3A	73.88 (3)
O15^viii^—Cs3A—O16^viii^	52.43 (7)	O16—Si6—Cs3B^xvi^	78.5 (2)
O16—Cs3A—O16^viii^	86.00 (8)	O13—Si6—Cs3B^xvi^	95.3 (3)
O6^viii^—Cs3A—O16^viii^	132.99 (8)	O3—Si6—Cs3B^xvi^	150.6 (2)
O1—Cs3A—Si2^viii^	143.72 (7)	O6^ix^—Si6—Cs3B^xvi^	45.60 (15)
O8^vii^—Cs3A—Si2^viii^	99.10 (6)	Cs3B^viii^—Si6—Cs3B^xvi^	134.4 (2)
O4^viii^—Cs3A—Si2^viii^	27.34 (5)	Cs3A^viii^—Si6—Cs3B^xvi^	132.72 (14)
O5^ix^—Cs3A—Si2^viii^	94.72 (6)	Cs3A—Si6—Cs3B^xvi^	83.8 (2)
O15^viii^—Cs3A—Si2^viii^	150.05 (8)	O16—Si6—Cs3A^xvi^	74.65 (12)
O16—Cs3A—Si2^viii^	72.93 (6)	O13—Si6—Cs3A^xvi^	101.69 (12)
O6^viii^—Cs3A—Si2^viii^	26.72 (5)	O3—Si6—Cs3A^xvi^	146.41 (14)
O16^viii^—Cs3A—Si2^viii^	111.97 (5)	O6^ix^—Si6—Cs3A^xvi^	45.44 (12)
O1—Cs3A—O5^viii^	122.52 (9)	Cs3B^viii^—Si6—Cs3A^xvi^	132.32 (15)
O8^vii^—Cs3A—O5^viii^	106.15 (7)	Cs3A^viii^—Si6—Cs3A^xvi^	129.68 (4)
O4^viii^—Cs3A—O5^viii^	45.90 (7)	Cs3A—Si6—Cs3A^xvi^	77.24 (3)
O5^ix^—Cs3A—O5^viii^	80.67 (8)	Cs3B^xvi^—Si6—Cs3A^xvi^	6.6 (2)
O15^viii^—Cs3A—O5^viii^	164.32 (7)	O17—Si7—O18	115.08 (18)
O16—Cs3A—O5^viii^	48.69 (8)	O17—Si7—O2	112.90 (17)
O6^viii^—Cs3A—O5^viii^	44.49 (7)	O18—Si7—O2	106.40 (17)
O16^viii^—Cs3A—O5^viii^	111.99 (7)	O17—Si7—O19^ix^	110.79 (16)
Si2^viii^—Cs3A—O5^viii^	25.63 (6)	O18—Si7—O19^ix^	104.08 (15)
O1—Cs3A—O3^viii^	92.49 (8)	O2—Si7—O19^ix^	106.91 (18)
O8^vii^—Cs3A—O3^viii^	143.14 (10)	O17—Si7—Cs1^ix^	124.89 (13)
O4^viii^—Cs3A—O3^viii^	43.96 (7)	O18—Si7—Cs1^ix^	57.65 (11)
O5^ix^—Cs3A—O3^viii^	158.91 (8)	O2—Si7—Cs1^ix^	121.55 (13)
O15^viii^—Cs3A—O3^viii^	85.90 (7)	O19^ix^—Si7—Cs1^ix^	46.64 (10)
O16—Cs3A—O3^viii^	85.25 (8)	O17—Si7—Cs2^iii^	49.62 (12)
O6^viii^—Cs3A—O3^viii^	89.48 (7)	O18—Si7—Cs2^iii^	66.05 (12)
O16^viii^—Cs3A—O3^viii^	43.53 (7)	O2—Si7—Cs2^iii^	121.72 (13)
Si2^viii^—Cs3A—O3^viii^	70.25 (5)	O19^ix^—Si7—Cs2^iii^	131.32 (13)
O5^viii^—Cs3A—O3^viii^	79.71 (7)	Cs1^ix^—Si7—Cs2^iii^	102.84 (3)
O1—Cs3A—Cs1^xiii^	55.38 (6)	O20—Si8—O21	111.19 (18)
O8^vii^—Cs3A—Cs1^xiii^	57.17 (6)	O20—Si8—O7	112.06 (18)
O4^viii^—Cs3A—Cs1^xiii^	172.38 (6)	O21—Si8—O7	110.13 (18)
O5^ix^—Cs3A—Cs1^xiii^	55.98 (5)	O20—Si8—O19	112.18 (16)
O15^viii^—Cs3A—Cs1^xiii^	56.64 (6)	O21—Si8—O19	106.10 (17)
O16—Cs3A—Cs1^xiii^	104.31 (6)	O7—Si8—O19	104.83 (18)
O6^viii^—Cs3A—Cs1^xiii^	127.54 (5)	O20—Si8—Cs1	125.41 (13)
O16^viii^—Cs3A—Cs1^xiii^	98.01 (5)	O21—Si8—Cs1	64.70 (12)
Si2^viii^—Cs3A—Cs1^xiii^	149.39 (3)	O7—Si8—Cs1	120.29 (13)
O5^viii^—Cs3A—Cs1^xiii^	135.18 (6)	O19—Si8—Cs1	41.45 (10)
O3^viii^—Cs3A—Cs1^xiii^	140.35 (5)	O20—Si8—Cs2^iii^	53.68 (12)
O15^viii^—Cs3B—O8^vii^	96.1 (3)	O21—Si8—Cs2^iii^	57.85 (13)
O15^viii^—Cs3B—O4^viii^	133.3 (4)	O7—Si8—Cs2^iii^	134.84 (13)
O8^vii^—Cs3B—O4^viii^	120.4 (3)	O19—Si8—Cs2^iii^	120.30 (13)
O15^viii^—Cs3B—O6^viii^	158.8 (6)	Cs1—Si8—Cs2^iii^	94.85 (3)
O8^vii^—Cs3B—O6^viii^	74.9 (2)	O20—Si8—Cs1^iv^	141.77 (13)
O4^viii^—Cs3B—O6^viii^	46.66 (13)	O21—Si8—Cs1^iv^	105.59 (14)
O15^viii^—Cs3B—O1	51.35 (16)	O7—Si8—Cs1^iv^	42.51 (12)
O8^vii^—Cs3B—O1	110.8 (3)	O19—Si8—Cs1^iv^	65.46 (12)
O4^viii^—Cs3B—O1	125.0 (4)	Cs1—Si8—Cs1^iv^	79.75 (2)
O6^viii^—Cs3B—O1	149.8 (6)	Cs2^iii^—Si8—Cs1^iv^	162.95 (3)
O15^viii^—Cs3B—O16^viii^	54.21 (15)	O20—Si8—Cs2	39.22 (11)
O8^vii^—Cs3B—O16^viii^	150.0 (3)	O21—Si8—Cs2	94.95 (13)
O4^viii^—Cs3B—O16^viii^	87.7 (2)	O7—Si8—Cs2	86.44 (12)
O6^viii^—Cs3B—O16^viii^	134.3 (3)	O19—Si8—Cs2	150.46 (11)
O1—Cs3B—O16^viii^	49.38 (16)	Cs1—Si8—Cs2	150.06 (4)
O15^viii^—Cs3B—O5^ix^	111.0 (2)	Cs2^iii^—Si8—Cs2	55.205 (18)
O8^vii^—Cs3B—O5^ix^	49.88 (16)	Cs1^iv^—Si8—Cs2	128.74 (3)
O4^viii^—Cs3B—O5^ix^	114.6 (4)	O22—Si9—O23	113.21 (14)
O6^viii^—Cs3B—O5^ix^	78.3 (2)	O22—Si9—O21	109.52 (18)
O1—Cs3B—O5^ix^	83.6 (3)	O23—Si9—O21	108.63 (19)
O16^viii^—Cs3B—O5^ix^	130.7 (5)	O22—Si9—O18	111.34 (17)
O15^viii^—Cs3B—O3^viii^	90.6 (2)	O23—Si9—O18	109.4 (2)
O8^vii^—Cs3B—O3^viii^	157.1 (5)	O21—Si9—O18	104.29 (17)
O4^viii^—Cs3B—O3^viii^	44.73 (12)	O22—Si9—Cs1	54.89 (11)
O6^viii^—Cs3B—O3^viii^	91.09 (19)	O23—Si9—Cs1	98.43 (16)
O1—Cs3B—O3^viii^	90.5 (2)	O21—Si9—Cs1	65.20 (12)
O16^viii^—Cs3B—O3^viii^	44.18 (12)	O18—Si9—Cs1	152.14 (13)
O5^ix^—Cs3B—O3^viii^	145.7 (5)	O22—Si9—Cs2^iii^	95.56 (13)
O15^viii^—Cs3B—Si2^viii^	159.9 (3)	O23—Si9—Cs2^iii^	150.37 (5)
O8^vii^—Cs3B—Si2^viii^	99.9 (2)	O21—Si9—Cs2^iii^	51.79 (13)
O4^viii^—Cs3B—Si2^viii^	26.56 (9)	O18—Si9—Cs2^iii^	63.31 (12)
O6^viii^—Cs3B—Si2^viii^	26.10 (8)	Cs1—Si9—Cs2^iii^	92.24 (2)
O1—Cs3B—Si2^viii^	131.0 (5)	O22—Si9—Cs1^ix^	78.16 (11)
O16^viii^—Cs3B—Si2^viii^	110.0 (2)	O23—Si9—Cs1^ix^	97.46 (16)
O5^ix^—Cs3B—Si2^viii^	88.7 (3)	O21—Si9—Cs1^ix^	145.97 (13)
O3^viii^—Cs3B—Si2^viii^	70.04 (14)	O18—Si9—Cs1^ix^	44.82 (11)
O15^viii^—Cs3B—O16	116.2 (4)	Cs1—Si9—Cs1^ix^	132.96 (3)
O8^vii^—Cs3B—O16	116.1 (4)	Cs2^iii^—Si9—Cs1^ix^	95.20 (2)
O4^viii^—Cs3B—O16	74.8 (3)	Si1—O1—Sn1^xvii^	131.57 (17)
O6^viii^—Cs3B—O16	84.9 (3)	Si1—O1—Cs3A	125.27 (15)
O1—Cs3B—O16	65.8 (3)	Sn1^xvii^—O1—Cs3A	100.12 (12)
O16^viii^—Cs3B—O16	79.0 (3)	Si1—O1—Cs1^xiii^	101.85 (14)
O5^ix^—Cs3B—O16	67.0 (3)	Sn1^xvii^—O1—Cs1^xiii^	108.13 (12)
O3^viii^—Cs3B—O16	79.8 (3)	Cs3A—O1—Cs1^xiii^	71.91 (6)
Si2^viii^—Cs3B—O16	66.8 (2)	Si1—O1—Cs3B	133.0 (3)
O15^viii^—Cs3B—Cs1^xiii^	57.45 (14)	Sn1^xvii^—O1—Cs3B	93.2 (3)
O8^vii^—Cs3B—Cs1^xiii^	57.53 (14)	Cs3A—O1—Cs3B	7.9 (2)
O4^viii^—Cs3B—Cs1^xiii^	167.5 (5)	Cs1^xiii^—O1—Cs3B	70.34 (15)
O6^viii^—Cs3B—Cs1^xiii^	126.7 (3)	Si1—O1—Cs3A^viii^	76.02 (12)
O1—Cs3B—Cs1^xiii^	53.63 (14)	Sn1^xvii^—O1—Cs3A^viii^	90.90 (10)
O16^viii^—Cs3B—Cs1^xiii^	97.3 (2)	Cs3A—O1—Cs3A^viii^	88.33 (8)
O5^ix^—Cs3B—Cs1^xiii^	53.71 (15)	Cs1^xiii^—O1—Cs3A^viii^	154.40 (9)
O3^viii^—Cs3B—Cs1^xiii^	141.5 (3)	Cs3B—O1—Cs3A^viii^	92.2 (2)
Si2^viii^—Cs3B—Cs1^xiii^	142.4 (4)	Si1—O2—Si7	144.3 (2)
O16—Cs3B—Cs1^xiii^	94.8 (4)	Si1—O2—Cs1^xiii^	90.29 (14)
O15^viii^—Cs3B—Si1^viii^	114.8 (2)	Si7—O2—Cs1^xiii^	112.69 (15)
O8^vii^—Cs3B—Si1^viii^	145.5 (3)	Si1—O3—Si6	159.1 (3)
O4^viii^—Cs3B—Si1^viii^	25.25 (8)	Si1—O3—Cs3B^viii^	85.1 (2)
O6^viii^—Cs3B—Si1^viii^	70.77 (15)	Si6—O3—Cs3B^viii^	88.02 (19)
O1—Cs3B—Si1^viii^	100.8 (3)	Si1—O3—Cs3A^viii^	80.57 (13)
O16^viii^—Cs3B—Si1^viii^	63.64 (15)	Si6—O3—Cs3A^viii^	90.22 (14)
O5^ix^—Cs3B—Si1^viii^	123.9 (4)	Cs3B^viii^—O3—Cs3A^viii^	7.7 (3)
O3^viii^—Cs3B—Si1^viii^	24.92 (7)	Si1—O4—Si2	129.51 (19)
Si2^viii^—Cs3B—Si1^viii^	46.82 (10)	Si1—O4—Cs3B^viii^	98.7 (2)
O16—Cs3B—Si1^viii^	64.7 (2)	Si2—O4—Cs3B^viii^	93.4 (2)
Cs1^xiii^—Cs3B—Si1^viii^	153.4 (4)	Si1—O4—Cs3A^viii^	95.66 (14)
O8—Sn1—O22^iii^	89.80 (12)	Si2—O4—Cs3A^viii^	89.04 (13)
O8—Sn1—O1^x^	177.68 (12)	Cs3B^viii^—O4—Cs3A^viii^	8.8 (3)
O22^iii^—Sn1—O1^x^	92.32 (13)	Si1—O4—Cs2	112.55 (14)
O8—Sn1—O15^vi^	95.43 (12)	Si2—O4—Cs2	114.54 (14)
O22^iii^—Sn1—O15^vi^	92.48 (12)	Cs3B^viii^—O4—Cs2	96.3 (3)
O1^x^—Sn1—O15^vi^	85.41 (12)	Cs3A^viii^—O4—Cs2	105.03 (9)
O8—Sn1—O16^vi^	91.37 (12)	Si2—O5—Sn1^xvii^	130.27 (17)
O22^iii^—Sn1—O16^vi^	176.09 (13)	Si2—O5—Cs3A^ii^	123.44 (14)
O1^x^—Sn1—O16^vi^	86.45 (12)	Sn1^xvii^—O5—Cs3A^ii^	106.29 (13)
O15^vi^—Sn1—O16^vi^	91.12 (12)	Si2—O5—Cs1^iv^	99.16 (13)
O8—Sn1—O5^x^	86.35 (12)	Sn1^xvii^—O5—Cs1^iv^	97.58 (10)
O22^iii^—Sn1—O5^x^	88.07 (12)	Cs3A^ii^—O5—Cs1^iv^	69.96 (6)
O1^x^—Sn1—O5^x^	92.80 (12)	Si2—O5—Cs3B^ii^	129.9 (3)
O15^vi^—Sn1—O5^x^	178.14 (12)	Sn1^xvii^—O5—Cs3B^ii^	99.8 (3)
O16^vi^—Sn1—O5^x^	88.28 (12)	Cs3A^ii^—O5—Cs3B^ii^	7.0 (2)
O8—Sn1—Cs3B^x^	122.9 (3)	Cs1^iv^—O5—Cs3B^ii^	67.68 (14)
O22^iii^—Sn1—Cs3B^x^	125.41 (13)	Si2—O5—Cs3A^viii^	76.70 (12)
O1^x^—Sn1—Cs3B^x^	56.3 (3)	Sn1^xvii^—O5—Cs3A^viii^	96.58 (10)
O15^vi^—Sn1—Cs3B^x^	47.0 (2)	Cs3A^ii^—O5—Cs3A^viii^	99.33 (8)
O16^vi^—Sn1—Cs3B^x^	56.66 (14)	Cs1^iv^—O5—Cs3A^viii^	164.22 (9)
O5^x^—Sn1—Cs3B^x^	131.4 (2)	Cs3B^ii^—O5—Cs3A^viii^	103.00 (19)
O8—Sn1—Cs3A^x^	129.10 (9)	Si2—O6—Si6^ii^	145.3 (2)
O22^iii^—Sn1—Cs3A^x^	125.11 (8)	Si2—O6—Cs3B^viii^	89.6 (2)
O1^x^—Sn1—Cs3A^x^	49.99 (9)	Si6^ii^—O6—Cs3B^viii^	113.8 (2)
O15^vi^—Sn1—Cs3A^x^	52.78 (8)	Si2—O6—Cs3A^viii^	85.23 (13)
O16^vi^—Sn1—Cs3A^x^	56.45 (9)	Si6^ii^—O6—Cs3A^viii^	114.18 (15)
O5^x^—Sn1—Cs3A^x^	125.54 (8)	Cs3B^viii^—O6—Cs3A^viii^	8.5 (3)
Cs3B^x^—Sn1—Cs3A^x^	7.0 (2)	Si2—O7—Si8	142.1 (2)
O8—Sn1—Cs1^iii^	54.30 (9)	Si2—O7—Cs1^iv^	98.93 (15)
O22^iii^—Sn1—Cs1^iii^	53.05 (8)	Si8—O7—Cs1^iv^	118.02 (15)
O1^x^—Sn1—Cs1^iii^	126.61 (9)	Si3—O8—Sn1	128.90 (17)
O15^vi^—Sn1—Cs1^iii^	128.78 (8)	Si3—O8—Cs3B^xiv^	115.3 (3)
O16^vi^—Sn1—Cs1^iii^	125.17 (9)	Sn1—O8—Cs3B^xiv^	115.8 (3)
O5^x^—Sn1—Cs1^iii^	52.84 (8)	Si3—O8—Cs3A^xiv^	123.52 (15)
Cs3B^x^—Sn1—Cs1^iii^	175.7 (2)	Sn1—O8—Cs3A^xiv^	107.58 (12)
Cs3A^x^—Sn1—Cs1^iii^	176.60 (2)	Cs3B^xiv^—O8—Cs3A^xiv^	8.6 (3)
O8—Sn1—Cs3A^xiv^	45.47 (8)	Si3—O8—Cs1^iii^	101.09 (13)
O22^iii^—Sn1—Cs3A^xiv^	106.03 (8)	Sn1—O8—Cs1^iii^	96.22 (12)
O1^x^—Sn1—Cs3A^xiv^	132.83 (9)	Cs3B^xiv^—O8—Cs1^iii^	72.34 (19)
O15^vi^—Sn1—Cs3A^xiv^	135.00 (9)	Cs3A^xiv^—O8—Cs1^iii^	69.61 (6)
O16^vi^—Sn1—Cs3A^xiv^	72.31 (9)	Si3—O9—Si5	175.2 (3)
O5^x^—Sn1—Cs3A^xiv^	46.38 (9)	Si4—O10—Si3	143.6 (2)
Cs3B^x^—Sn1—Cs3A^xiv^	128.32 (11)	Si4—O10—Cs1^iii^	108.30 (14)
Cs3A^x^—Sn1—Cs3A^xiv^	128.760 (13)	Si3—O10—Cs1^iii^	103.11 (14)
Cs1^iii^—Sn1—Cs3A^xiv^	52.982 (8)	Si4—O10—Cs2	90.03 (13)
O8—Sn1—Cs1^xi^	136.49 (8)	Si3—O10—Cs2	92.47 (13)
O22^iii^—Sn1—Cs1^xi^	72.06 (8)	Cs1^iii^—O10—Cs2	115.94 (9)
O1^x^—Sn1—Cs1^xi^	45.31 (8)	Si5^vi^—O11—Si3	130.59 (18)
O15^vi^—Sn1—Cs1^xi^	48.21 (9)	Si5^vi^—O11—Cs2	108.23 (14)
O16^vi^—Sn1—Cs1^xi^	109.41 (9)	Si3—O11—Cs2	116.67 (14)
O5^x^—Sn1—Cs1^xi^	130.44 (9)	Si4—O12—Sn2^ix^	164.45 (19)
Cs3B^x^—Sn1—Cs1^xi^	53.90 (11)	Si4—O12—Cs2	100.26 (13)
Cs3A^x^—Sn1—Cs1^xi^	53.058 (9)	Sn2^ix^—O12—Cs2	94.97 (10)
Cs1^iii^—Sn1—Cs1^xi^	125.031 (10)	Si6—O13—Si4	138.7 (2)
Cs3A^xiv^—Sn1—Cs1^xi^	176.69 (2)	Si5—O14—Si4^vi^	139.6 (2)
O8—Sn1—Cs3A^vi^	116.76 (8)	Si5—O14—Cs2^xii^	99.13 (13)
O22^iii^—Sn1—Cs3A^vi^	130.18 (9)	Si4^vi^—O14—Cs2^xii^	114.59 (14)
O1^x^—Sn1—Cs3A^vi^	61.07 (9)	Si5—O14—Cs1^xv^	92.19 (14)
O15^vi^—Sn1—Cs3A^vi^	123.03 (8)	Si4^vi^—O14—Cs1^xv^	88.28 (13)
O16^vi^—Sn1—Cs3A^vi^	46.12 (9)	Cs2^xii^—O14—Cs1^xv^	122.15 (10)
O5^x^—Sn1—Cs3A^vi^	55.46 (8)	Si5—O15—Sn1^xii^	129.25 (17)
Cs3B^x^—Sn1—Cs3A^vi^	76.1 (2)	Si5—O15—Cs3B^viii^	120.2 (4)
Cs3A^x^—Sn1—Cs3A^vi^	70.79 (2)	Sn1^xii^—O15—Cs3B^viii^	103.3 (3)
Cs1^iii^—Sn1—Cs3A^vi^	108.007 (10)	Si5—O15—Cs3A^viii^	128.03 (16)
Cs3A^xiv^—Sn1—Cs3A^vi^	74.36 (3)	Sn1^xii^—O15—Cs3A^viii^	97.02 (10)
Cs1^xi^—Sn1—Cs3A^vi^	104.687 (12)	Cs3B^viii^—O15—Cs3A^viii^	8.1 (3)
O12^iii^—Sn2—O12^ii^	180.00 (18)	Si5—O15—Cs1^xv^	111.68 (14)
O12^iii^—Sn2—O20	88.36 (12)	Sn1^xii^—O15—Cs1^xv^	104.69 (12)
O12^ii^—Sn2—O20	91.64 (12)	Cs3B^viii^—O15—Cs1^xv^	73.1 (2)
O12^iii^—Sn2—O20^xi^	91.64 (12)	Cs3A^viii^—O15—Cs1^xv^	69.45 (6)
O12^ii^—Sn2—O20^xi^	88.36 (12)	Si6—O16—Sn1^xii^	139.84 (18)
O20—Sn2—O20^xi^	180.0	Si6—O16—Cs3A	108.61 (13)
O12^iii^—Sn2—O17^iii^	90.26 (12)	Sn1^xii^—O16—Cs3A	107.16 (12)
O12^ii^—Sn2—O17^iii^	89.74 (12)	Si6—O16—Cs3B^viii^	97.1 (3)
O20—Sn2—O17^iii^	85.77 (12)	Sn1^xii^—O16—Cs3B^viii^	92.74 (16)
O20^xi^—Sn2—O17^iii^	94.23 (12)	Cs3A—O16—Cs3B^viii^	102.1 (3)
O12^iii^—Sn2—O17^ii^	89.74 (12)	Si6—O16—Cs3A^viii^	101.94 (14)
O12^ii^—Sn2—O17^ii^	90.26 (12)	Sn1^xii^—O16—Cs3A^viii^	93.15 (10)
O20—Sn2—O17^ii^	94.23 (12)	Cs3A—O16—Cs3A^viii^	94.00 (8)
O20^xi^—Sn2—O17^ii^	85.77 (12)	Cs3B^viii^—O16—Cs3A^viii^	8.4 (3)
O17^iii^—Sn2—O17^ii^	180.00 (18)	Si6—O16—Cs3B	110.77 (18)
O12^iii^—Sn2—Cs2^iii^	55.33 (8)	Sn1^xii^—O16—Cs3B	105.33 (17)
O12^ii^—Sn2—Cs2^iii^	124.67 (8)	Cs3A—O16—Cs3B	2.28 (13)
O20—Sn2—Cs2^iii^	53.35 (9)	Cs3B^viii^—O16—Cs3B	101.0 (3)
O20^xi^—Sn2—Cs2^iii^	126.65 (9)	Cs3A^viii^—O16—Cs3B	92.79 (14)
O17^iii^—Sn2—Cs2^iii^	51.61 (8)	Si7—O17—Sn2^ix^	130.26 (17)
O17^ii^—Sn2—Cs2^iii^	128.39 (8)	Si7—O17—Cs2	125.86 (15)
O12^iii^—Sn2—Cs2^ii^	124.67 (8)	Sn2^ix^—O17—Cs2	98.65 (10)
O12^ii^—Sn2—Cs2^ii^	55.33 (8)	Si7—O17—Cs2^iii^	108.81 (14)
O20—Sn2—Cs2^ii^	126.65 (9)	Sn2^ix^—O17—Cs2^iii^	104.82 (12)
O20^xi^—Sn2—Cs2^ii^	53.35 (9)	Cs2—O17—Cs2^iii^	72.17 (6)
O17^iii^—Sn2—Cs2^ii^	128.39 (8)	Si7—O18—Si9	137.1 (2)
O17^ii^—Sn2—Cs2^ii^	51.61 (8)	Si7—O18—Cs1^ix^	97.47 (13)
Cs2^iii^—Sn2—Cs2^ii^	180.0	Si9—O18—Cs1^ix^	114.47 (14)
O12^iii^—Sn2—Cs2	109.74 (8)	Si7—O18—Cs2^iii^	90.66 (13)
O12^ii^—Sn2—Cs2	70.26 (8)	Si9—O18—Cs2^iii^	93.76 (13)
O20—Sn2—Cs2	45.77 (8)	Cs1^ix^—O18—Cs2^iii^	124.26 (10)
O20^xi^—Sn2—Cs2	134.23 (8)	Si8—O19—Si7^ii^	129.52 (18)
O17^iii^—Sn2—Cs2	47.71 (9)	Si8—O19—Cs1	117.12 (14)
O17^ii^—Sn2—Cs2	132.29 (9)	Si7^ii^—O19—Cs1	109.67 (13)
Cs2^iii^—Sn2—Cs2	54.535 (8)	Si8—O20—Sn2	129.48 (17)
Cs2^ii^—Sn2—Cs2	125.465 (8)	Si8—O20—Cs2	122.70 (15)
O12^iii^—Sn2—Cs2^xi^	70.26 (8)	Sn2—O20—Cs2	107.37 (11)
O12^ii^—Sn2—Cs2^xi^	109.74 (8)	Si8—O20—Cs2^iii^	103.61 (14)
O20—Sn2—Cs2^xi^	134.23 (8)	Sn2—O20—Cs2^iii^	97.05 (12)
O20^xi^—Sn2—Cs2^xi^	45.77 (8)	Cs2—O20—Cs2^iii^	72.15 (6)
O17^iii^—Sn2—Cs2^xi^	132.29 (9)	Si8—O21—Si9	151.9 (3)
O17^ii^—Sn2—Cs2^xi^	47.71 (9)	Si8—O21—Cs2^iii^	98.65 (14)
Cs2^iii^—Sn2—Cs2^xi^	125.466 (8)	Si9—O21—Cs2^iii^	106.27 (15)
Cs2^ii^—Sn2—Cs2^xi^	54.534 (8)	Si8—O21—Cs1	91.31 (13)
Cs2—Sn2—Cs2^xi^	180.0	Si9—O21—Cs1	90.47 (13)
O1—Si1—O2	110.44 (18)	Cs2^iii^—O21—Cs1	113.55 (10)
O1—Si1—O3	111.15 (18)	Si9—O22—Sn1^iii^	160.20 (19)
O2—Si1—O3	108.86 (18)	Si9—O22—Cs1	102.17 (14)
O1—Si1—O4	111.33 (16)	Sn1^iii^—O22—Cs1	97.49 (10)
O2—Si1—O4	108.37 (18)	Si9^iv^—O23—Si9	179.5 (3)
O3—Si1—O4	106.57 (18)		

Symmetry codes: (i) −*x*+1, *y*+1, −*z*+1/2; (ii) *x*, *y*+1, *z*; (iii) −*x*+1, −*y*−1, −*z*; (iv) −*x*+1, *y*, −*z*+1/2; (v) *x*−1/2, −*y*−1/2, *z*; (vi) −*x*+3/2, *y*+1/2, −*z*; (vii) *x*, −*y*−2, *z*+1/2; (viii) −*x*+3/2, −*y*−3/2, −*z*+1/2; (ix) *x*, *y*−1, *z*; (x) *x*, −*y*−1, *z*−1/2; (xi) −*x*+1, −*y*, −*z*; (xii) −*x*+3/2, *y*−1/2, −*z*; (xiii) −*x*+1, *y*−1, −*z*+1/2; (xiv) *x*, −*y*−2, *z*−1/2; (xv) *x*+1/2, −*y*−1/2, *z*; (xvi) −*x*+3/2, −*y*−5/2, −*z*+1/2; (xvii) *x*, −*y*−1, *z*+1/2.
